# Short-term Impact of Mass Drug Administration With Dihydroartemisinin Plus Piperaquine on Malaria in Southern Province Zambia: A Cluster-Randomized Controlled Trial

**DOI:** 10.1093/infdis/jiw416

**Published:** 2016-12-05

**Authors:** Thomas P Eisele, Adam Bennett, Kafula Silumbe, Timothy P Finn, Victor Chalwe, Mulakwa Kamuliwo, Busiku Hamainza, Hawela Moonga, Emmanuel Kooma, Elizabeth Chizema Kawesha, Joshua Yukich, Joseph Keating, Travis Porter, Ruben O Conner, Duncan Earle, Richard W Steketee, John M Miller

**Affiliations:** 1 Center for Applied Malaria Research and Evaluation, Department of Tropical Medicine, Tulane University School of Public Health and Tropical Medicine, New Orleans, Louisiana; 2 Malaria Elimination Initiative, Global Health Group, University of California San Francisco; 3 PATH-MACEPA, Seattle, Washington; 4 PATH-Malaria Control and Elimination Partnership in Africa (MACEPA), National Malaria Control Centre, Chainama Hospital College Grounds; 5 Institute for Medical Research and Training, University Teaching Hospital; 6 National Malaria Control Centre, Zambia Ministry of Health, Chainama Hospital, Lusaka; 7 Zambia Ministry of Health, Southern Provincial Health Office, Choma

**Keywords:** malaria, elimination, mass drug administration

## Abstract

**Background:**

Mass drug administration (MDA) using dihydroartemisinin plus piperaquine (DHAp) represents a potential strategy to clear *Plasmodium falciparum* infections and reduce the human parasite reservoir.

**Methods:**

A cluster-randomized controlled trial in Southern Province, Zambia, was used to assess the short-term impact of 2 rounds of community-wide MDA and household-level (focal) MDA with DHAp compared with no mass treatment. Study end points included parasite prevalence in children, infection incidence, and confirmed malaria case incidence.

**Results:**

All end points significantly decreased after intervention, irrespective of treatment group. Parasite prevalence from 7.71% at baseline to 0.54% after MDA in lower-transmission areas, resulting in an 87% reduction compared with control (adjusted odds ratio, 0.13; 95% confidence interval, .02–.92;*P* = .04). No difference between treatment groups was observed in areas of high transmission. The 5-month cumulative infection incidence was 70% lower (crude incidence rate ratio, 0.30; 95% confidence interval, .06–1.49; *P* = .14) and 58% lower (0.42; .18–.98;*P* = .046) after MDA compared with control in lower- and higher-transmission areas, respectively. No significant impact of focal MDA was observed for any end point.

**Conclusions:**

Two rounds of MDA with DHAp rapidly reduced infection prevalence, infection incidence, and confirmed case incidence rates, especially in low-transmission areas.

**Clinical Trials Registration:**

NCT02329301.

**(see editorial commentary by Chen et al on pages 1790–2.)**

Over the past decade, Zambia has successfully scaled up the World Health Organization–recommended malaria control interventions (eg, long-lasting insecticide-treated nets, indoor residual spraying, and prompt effective diagnosis and treatment) and is considering alternative strategies to further reduce the malaria burden [1]. Although it is recognized that malaria elimination will take time to achieve, elimination is considered a realistic goal in Zambia in light of recent successes against malaria [2].

Mass testing and treatment interventions, wherein individuals are tested with a rapid diagnostic test (RDT) and treated if results are positive, have had limited impact on malaria in Southern Zambia and elsewhere [3–7], primarily because RDTs miss many low-density parasite infections [8–10]. When combined with universal coverage of vector control, good access to case management and strong surveillance, mass drug administration (MDA) (ie, where everyone in a target area is treated with a long-acting antimalarial, such as dihydroartemisinin plus piperaquine [DHAp] [11–14]) may be a potential strategy to shorten Zambia's timeline toward elimination. Focal MDA (fMDA) is similar but provides presumptive treatment to household members only when ≥1 resident is confirmed positive by RDT.

The primary goal of using 2 rounds of MDA or fMDA with DHAp in Zambia is to reduce the malaria parasite infection prevalence and incidence to preelimination levels during peak transmission in low-transmission areas [15] and to halve prevalence and incidence in high-transmission areas. It is then anticipated that such gains can be sustained by strong surveillance, community case management, and universal coverage of vector control. In this context, we present results from a trial aimed at quantifying the relative effectiveness of 2 rounds of MDA and fMDA with DHAp, against no mass treatment, for reducing malaria infection prevalence and incidence in Southern Province, Zambia. The follow-up period presented here is for 5 months during the high-transmission season after the first mass treatment round. Although additional mass treatment rounds and longer-term follow-up are ongoing, measures of short-term impact of the first 2 rounds are important to ensure these strategies can significantly reduce malaria in this context. If impact is shown, less aggressive strategies could be used to sustain the gains in the longer term, with the goal of eventual elimination from this area.

## METHODS

### Protocol

The full protocol for this trial has been published elsewhere [16]. Ethical approval was obtained from Tulane University, Western Institutional Review Board, the University of Zambia, and the Zambia Medicines Regulatory Authority. Informed consent was obtained from all enrolled subjects. Written informed consent (or assent for those ≥6 and <18 years old) was obtained from each participant before enrollment.

### Study Design and Participants

**Figure 1.**
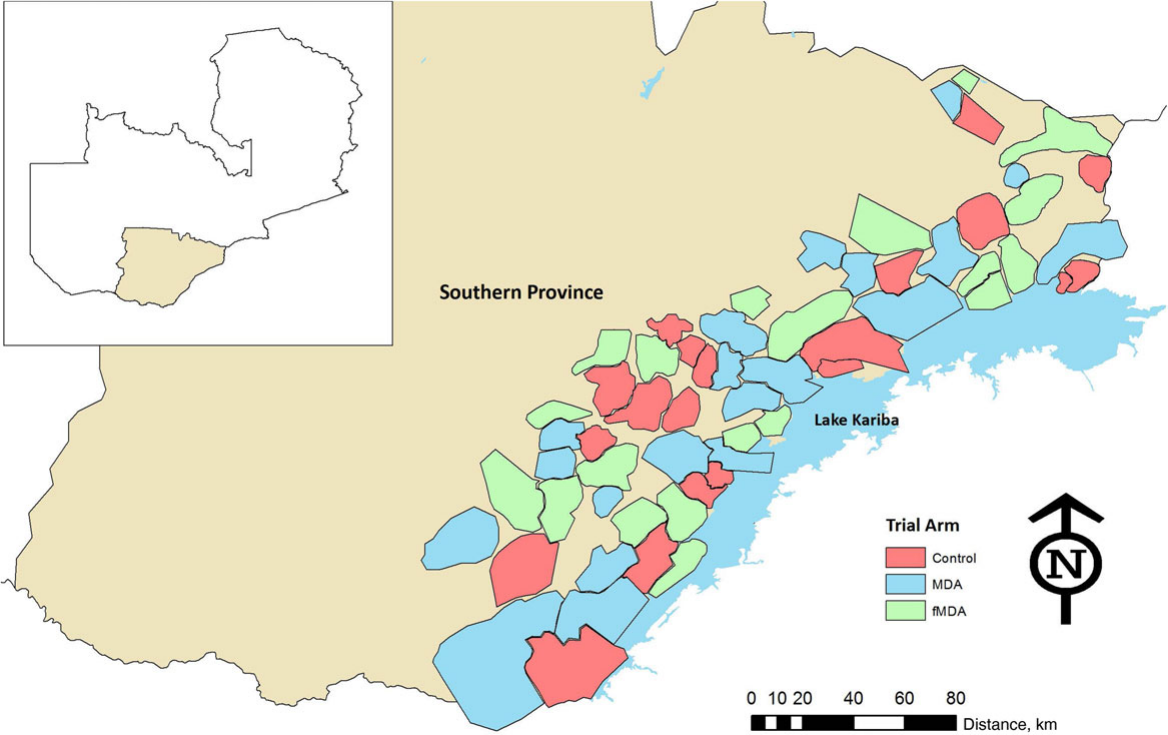
Map of the study area, divided into 60 health facility catchment areas that served as the unit of randomization. Abbreviations: fMDA, focal mass drug administration; MDA, mass drug administration; N, north.

A cluster-randomized controlled trial (CRCT) was used to evaluate the impact of the mass treatment interventions on study end points. The trial area was stratified into higher- and lower-transmission strata above and below 10% parasite prevalence at randomization. The study was conducted in Southern Province, Zambia along Lake Kariba in 60 health facility catchment areas (HFCAs) in 10 districts (Figure 1). The entire study area was enumerated by a geographic information system in 2013 and 2014 using personal digital assistants. Approximately 330 000 individuals in 56 000 households, primarily of the Tonga ethnic group, live in this area. Malaria parasite prevalence in children ranges from <1% in areas inland from the lake to >25% in areas closer to the lake. The season for high malaria transmission lasts from January to May, coinciding with seasonal rains.

### Randomization

**Figure 2.**
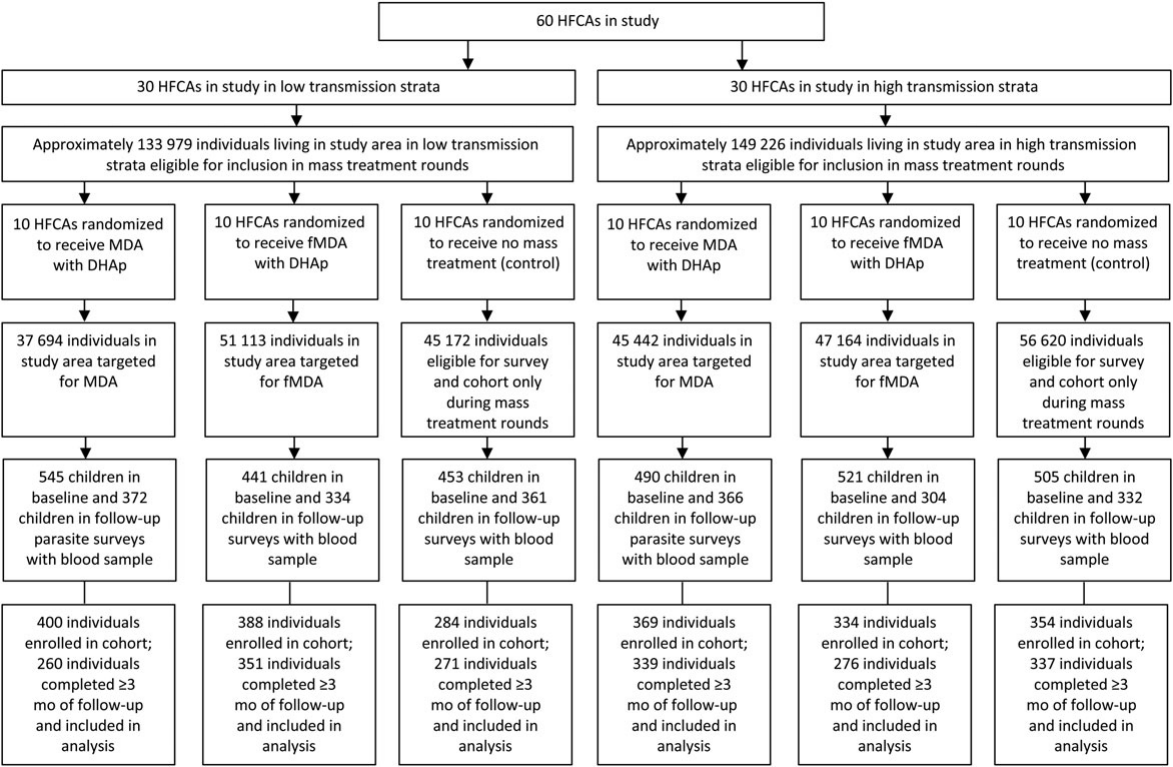
Trial profile. Sixty health facility catchment areas (HFCAs) served as the unit of randomization. After matching for transmission stratum and HFCA population size, HFCAs were randomly assigned to either mass drug administration (MDA), focal MDA (fMDA), or control groups, using the random allocation rule. As a result, 10 HFCAs per transmission stratum were assigned to MDA, fMDA, and control groups. All eligible participants in MDA and fMDA HFCAs each received 2 rounds of these mass treatment interventions. The primary end point of infection prevalence was measured before and after the mass treatment rounds with a parasite survey. The secondary end point of cumulative infection incidence was measured with a prospective cohort, with 5 months of follow-up after round 1 of the mass treatment rounds. Individuals in control HFCAs did not receive any mass treatment but were eligible to participate in the parasite surveys and cohort. Abbreviation: DHAp, dihydroartemisinin plus piperaquine.

HFCAs served as the unit of randomization (Figure 2). After stratification by transmission and HFCA population size, 60 HFCAs were randomly assigned to the MDA, fMDA, or control group using the random allocation rule, resulting in 10 HFCAs per transmission stratum in MDA, fMDA, and control groups. Allocation of intervention could not be blinded.

### Interventions

The entire study area received the standard of care in Zambia irrespective of treatment group, which consists of diagnosing all suspected cases presenting to the health system with either an RDT or microscopy and treating all individuals with positive results with the first-line drug artemether-lumefantrine (AL) [17]. Household coverage rates for long-lasting insecticide-treated nets and indoor residual spraying were 85% and 30%, respectively, in the study area. Since 2014, Zambia has scaled up community diagnosis and case management in Southern Province, including reactive case detection in areas with manageable case counts, representing an enhanced standard of care across the entire study site.

DHAp (Eurartesim; Sigma-Tau) was used presumptively to treat *Plasmodium falciparum* infections during the mass treatment rounds. All individuals meeting the inclusion criteria to receive DHAp were offered an age-appropriate 3-day course of DHAp based on manufacturer's recommendations and national treatment guidelines [17]. The first and last courses were given as directly observed therapy by the study team.

All individuals in intervention clusters were tested for parasite infections using an RDT (SD Bioline Malaria Antigen P.f test for detecting histidine-rich protein 2 antigen) during each of the 2 house-to-house mass treatment rounds. MDA consisted of offering all eligible individuals DHAp, irrespective of RDT result (Supplementary Figure 1). fMDA consisted of offering DHAp to all eligible individuals who resided in a household where anyone tested positive by RDT (Supplementary Figure 2). The control group received the standard of care, described above, but did not receive any mass treatment intervention.

Children <3 months old and pregnant women in their first trimester were excluded from receiving DHAp, according to the manufacturer's recommendations; they were instead offered the appropriate dose of antimalarial treatment according to Zambian national policy if RDT positive (Supplementary Figures 1 and 2). All individuals with suspected severe malaria or other severe illness were referred to the nearest health facility and omitted from the study.

### Study End Points

The primary end point was malaria parasite infection prevalence among children ≥3 months to <6 years old, defined as the proportion of children with a malaria parasite infection by RDT. Secondary end points included the cumulative infection incidence rate among all persons ≥3 months old (No. of RDT parasite infections in a prospective cohort divided by total time of exposure during 5-month follow-up [January–May 2015]), and the confirmed malaria case incidence rate (No. of outpatient laboratory-confirmed malaria cases for all ages per 1000 population per year).

### Procedures

#### Malaria Parasite Infection Prevalence

Malaria parasite infection prevalence in children was measured by a simple random sample of households during the high-transmission season (April–May) before the mass treatment rounds in 2014 (baseline) and again after the mass treatment rounds in 2015 (follow-up). A sample size of 2820 children at each survey round was required to detect a 50% reduction in infection prevalence with 80% power taking into account the cluster randomization, as described elsewhere [16]. RDTs and microscopy were used to assess the malaria parasite infection status among included children. RDT-positive children were treated with AL, according to national guidelines [17].

#### Cumulative Infection Incidence

The cumulative infection incidence rate was measured in individuals enrolled in a prospective cohort followed from December 2014 through the end of May 2015 (Supplementary Figure 3). A target sample size of 2250 individuals was sought to detect a 50% reduction in infection incidence between either mass treatment group and control, with 80% statistical power taking into account the cluster randomization, as described elsewhere [16]. Cohort participants were drawn from a simple random sample within each HFCA (39 persons within 13 households in each HFCA). The start of the cohort coincided with round 1 of the mass treatment interventions. Individuals enrolled from MDA and fMDA HFCAs received those interventions; all RDT-positive individuals in the control group were cleared of their malaria parasite infection at enrollment using AL [17]. All individuals in the cohort were followed up monthly with RDTs and microscopy. All RDT-positive individuals at monthly follow-up visits were treated with AL, except during the second mass treatment round for those in MDA and fMDA groups.

#### Confirmed Malaria Case Incidence

Routine data from the health management information system on monthly laboratory-confirmed outpatient malaria cases were ascertained from all 60 healthcare facilities in the study area from January 2011 onward. Confirmed case counts were standardized by the estimated midyear populations of each HFCA to obtain the incidence per 1000 population.

### Statistical Analysis

The CRCT study design was accounted for by including the cluster (HFCA) as a random effect in all analyses. All analyses were intention-to-treat analyses wherein all individuals were assumed to receive the treatment assigned to their HFCA at randomization. The treatment effect between the MDA/fMDA and control group for malaria infection prevalence in children was estimated for the 2015 follow-up (posttest) survey time point, using a crude odds ratio (OR) in a bivariate logistic regression model. A secondary model, defined a priori, adjusted for child age (in years), sex, household wealth, rainfall, the enhanced vegetation index, household elevation, and household protection vector control.

Crude incidence rate ratios (IRRs) were used to estimate the treatment effect of the MDA/fMDA on the cumulative infection incidence, compared with the control group, using a negative binomial model. Individuals present at enrollment that completed ≥3 months of follow-up were included in the analysis.

The treatment effect of MDA/fMDA on the confirmed malaria case incidence, compared with the control group, was estimated using a negative binomial model, standardized by midyear HFCA population. The model controlled for previous month's cases, calendar month, and anomalies in monthly rainfall and enhanced vegetation index. The baseline preintervention period was January–May 2013 and January–May 2014, and the follow-up postintervention period was January–May 2015. Because there were significant differences across treatment groups in the monthly confirmed malaria case incidence during the baseline period, a difference-in-differences model was used to account for baseline differences.

## RESULTS

Rounds 1 and 2 of the MDA and fMDA were successfully implemented in December 2014 and February–March 2015, respectively. Based on the 2015 follow-up survey, household coverage by MDA teams was 88.1% and 72.0% for rounds 1 and 2, respectively, and 62.5% and 54.0% for fMDA, respectively (Supplementary Table 1). The rate of adherence to a full course of DHAp was >85%, and the rate of refusal to partake in the study was very low across treatment rounds at <1%. Monthly total rainfall amounts during the study period were similar between treatment groups (see Supplementary Figures 4 and 5).

### Malaria Infection Prevalence

The baseline parasite survey conducted from April–May 2014 before the mass treatment interventions showed child infection prevalence, child and household demographics, treatment seeking for fevers, intervention coverage, and climate to be similar across treatment groups (Tables 1 and 2). There were significant declines in infection prevalence after the mass treatment rounds (follow-up survey conducted April–May 2015), irrespective of treatment group, transmission stratum, or diagnostic method (Table 2 and Supplementary Table 2). The largest proportional decline was observed among children in the MDA group in the lower-transmission setting, in whom infection prevalence decreased from 7.71% (42 infections among 545 children) at baseline to 0.54% (2 infections among 372 children) after MDA (93% decline). This represents a marginally significant relative reduction of 81% in the crude OR for having an infection compared with the control group (crude OR, 0.19; 95% confidence interval [CI], .29–1.28;*P* = .09), and a statistically significant 87% relative reduction (adjusted OR, 0.13; 95% CI, .02–.92; *P* = .04) after accounting for confounding factors. No other significant differences were observed.

**Table 1.** Baseline Characteristics of Intervention and Control Households Obtained From the Parasite Survey in April–May 2014

Characteristic	Mean Value (95% CI)		
	MDA (1047 Children/857 Households)	fMDA (985 Children/850 Households)	Control (976 Children/866 Households)
Age of children included for parasite testing, %			
3 mo to <6 y	12.70 (9.81–15.60)	11.98 (9.98–14.03)	12.81 (10.62–14.50)
1 y	17.96 (15.89–20.02)	17.87 (14.73–21.01)	15.68 (13.56–17.79)
2 y	15.85 (13.99–17.72)	17.36 (15.18–19.54)	17.52 (13.45–21.59)
3 y	15.76 (13.44–18.07)	17.46 (14.95–19.97)	17.62 (15.29–19.96)
4 y	17.67 (14.50–20.84)	17.87 (15.28–20.45)	19.06 (17.34–20.77)
5 y	20.06 (17.23–22.88)	17.46 (14.70–20.23)	17.32 (14.08–20.55)
Sex of children included for parasite testing, % male	48.14 (44.90–51.37)	50.86 (46.44–55.28)	52.25 (49.00–55.51)
Children with fever in the past 2 wk taken for treatment at a public or private provider, %	60.55 (48.15–72.95)	67.74 (59.94–75.55)	69.20 (61.34–77.06)
Children included for parasite testing by household wealth quintile, %			
1 (poorest)	32.66 (23.63–41.70)	21.73 (15.63–27.82)	27.97 (20.05–35.89)
2	19.10 (13.38–24.82)	25.69 (20.78–30.59)	19.06 (14.41–23.70)
3	20.73 (15.77–25.68)	19.29 (14.21–24.37)	21.21 (16.16–26.26)
4	17.86 (12.64–23.08)	16.65 (12.20–21.10)	18.95 (14.38–23.53)
5 (least poor)	9.65 (6.04–13.25)	16.65 (9.41–23.89)	12.81 (6.99–18.63)
Households with ≥1 LLIN, %	70.25 (62.44–78.05)	73.18 (65.78–80.57)	75.29 (68.71–81.87)
Households with IRS in past 12 mo, %^a^	6.88 (2.34–11.43)	19.65 (9.46–29.84)	16.86 (6.13–27.59)
Total rainfall for February–March 2014, mm	117.87 (106.64–129.11)	116.25 (106.30–126.19)	117.25 (107.56–126.93)
EVI for February–March 2014	0.43 (.41–.45)	0.41 (.39–.44)	0.42 (.41–.44)
Elevation, m	853.62 (708.34–998.76)	840.88 (670.00–1011.77)	819.48 (659.25–979.71)

Abbreviations: CI, confidence interval; EVI, enhanced vegetation index; fMDA, focal mass drug administration; IRS, indoor residual spraying; LLIN, long-lasting insecticide-treated net; MDA, mass drug administration.

^a^ Significantly different at *P* < .05.

**Table 2.** Baseline and Follow-Up Malaria RDT-Based Parasite Infection Prevalence Among Children <6 Years Old, as Measured by Baseline and Follow-Up Household Surveys During Peak Malaria Transmission Season (April–May 2014 and 2015)^a^

Treatment Group	Baseline (April–May 2014)				Follow-up (April–May 2015)				
	Children, Tested No.	Positive Results, No.	Positive Results, % (95% CI)	Crude OR vs Control (95% CI)	Children Tested, No.	Positive Results,	Positive Results, % (95% CI)	Crude OR vs Control (95% CI)	Adjusted OR (95% CI)^b^
Lower-transmission stratum									
MDA	545	42	7.71 (2.13–12.28)	0.87 (.31–2.43)	372	2	0.54 (.00–1.74)	0.19 (.03–1.28)^c^	0.13 (.02–.92)^d^
fMDA	441	39	8.84 (1.88–15.80)	0.99 (.36–2.79)	334	4	1.20 (.00–2.79)	0.49 (.11–2.29)	0.57 (.13–2.50)
Control	453	42	9.27 (3.11–15.44)	Reference	361	9	2.49 (.21–4.78)	Reference	Reference
Higher-transmission stratum									
MDA	490	248	50.61 (35.40–63.38)	0.68 (.28–1.66)	366	56	15.30 (4.68–25.92)	0.93 (.26–3.35)	0.86 (.25–3.04)
fMDA	521	270	51.82 (36.02–67.63)	0.72 (.30–1.74)	304	47	15.46 (5.08–25.84)	1.07 (.30–3.86)	1.28 (.36–4.60)
Control	505	283	56.04 (38.60–73.48)	Reference	332	55	16.57 (7.87–25.26)	Reference	Reference

Abbreviations: CI, confidence interval; fMDA, focal mass drug administration; MDA, mass drug administration; OR, odds ratio; RDT, rapid diagnostic test.

^a^ All standard errors of treatment effects are adjusted to account for the cluster-randomized controlled trial study design using a random effect at the cluster level. Children included were aged 3–71 mo.

^b^ Model adjusted for child age, sex, household wealth, rainfall, enhanced vegetation index, household elevation, and household protection by long-lasting insecticide-treated nets and indoor residual spraying.

^c^ *P* < .10.

^d^ *P* < .05.

### Cumulative Infection Incidence

A total of 2138 individuals were enrolled into the cohort from 1 December 2014 to 16 January 2015 and followed up through 13 June 2015 (Figure 2 and Supplementary Figure 3). A total of 1834 individuals completed ≥3 months of follow-up and were included in the cohort analysis, resulting in a loss-to-follow-up rate of 14.22%. Data among individuals enrolled but with <3 months of follow-up (lost to follow-up) showed them to be similar in age and baseline parasite prevalence; individuals lost to follow-up in the control group were younger than either mass treatment group, and more were male. During the 5-month follow-up period after round 1 of the mass treatment intervention in the lower-transmission stratum, individuals receiving MDA had a cumulative infection incidence of 3.44 per 1000 person-months (95% CI, .94–8.81) compared with 18.71 (11.72–28.32) in control areas, representing a nonsignificant relative reduction of 70% (crude IRR, 0.30; 95% CI, .06–1.49; *P* = .14) (Table 3). In the higher-transmission stratum, individuals receiving MDA had a cumulative infection incidence of 35.69 per 1000 person-months (95% CI, 26.89–46.46) compared with 91.27 (76.63–107.90) in control areas, representing a significant relative reduction of 58% (crude IRR, 0.42; 95% CI, .18–.98; *P* = .046). No other significant differences were observed.

**Table 3.** Cumulative Malaria Infection Incidence by RDT Among Individuals ≥3 Months Old in Cohort Households Followed Up Monthly From December 2014 to May 2015^a^

Treatment Group	Total Person-Months	Positive Results, No.	Cumulative IR/1000 Person-Months (95% CI)	Crude IRR vs Control (95% CI)
Lower-transmission stratum				
MDA	1163	4	3.44 (.94–8.81)	0.30 (.06–1.49)
fMDA	1616	19	11.76 (7.08–18.36)	0.77 (.22–2.71)
Control	1176	22	18.71 (11.72–28.32)	Reference
Higher-transmission stratum				
MDA	1541	55	35.69 (26.88–46.46)	0.41 (.18–.98)^b^
fMDA	1255	79	62.95 (49.84–78.45)	0.75 (.31–1.78)
Control	1501	137	91.27 (76.63–107.89)	Reference

Abbreviations: CI, confidence interval; fMDA, focal mass drug administration; IR, incidence rate; IRR, IR ratio; MDA, mass drug administration; RDT, rapid diagnostic test.

^a^ All standard errors of treatment effects are adjusted to account for the cluster-randomized controlled trial study design, using a random effect at the cluster level.

^b^ *P* < .05.

### Confirmed Malaria Case Incidence

Large declines in the monthly confirmed malaria case incidence rates were observed after the mass treatment rounds, including in the control group (Table 4). Among HFCAs in lower-transmission areas, the monthly confirmed case incidence declined >3-fold after 2 rounds of MDA, from 7.45 to 2.23 cases per 1000 HFCA population, representing a significantly (50%) larger decline than observed in the control group (difference-in-differences IRR, 0.50; 95% CI, .35–.72;*P* < .01). No other significant differences were observed.

**Table 4.** Mean Monthly Confirmed Malaria Case Incidence From the Routine Health Information System^a^

Treatment Group	Monthly Confirmed Malaria Case Incidence, Cases/1000 HFCA Population		Difference-in-Differences IRR (95% CI)
	Preintervention Period (January–May 2013 and 2014)	Postintervention Period (January–May 2015)	
Lower-transmission stratum			
MDA	7.45	2.23	0.50 (.35–.72)^b^
fMDA	13.54	7.78	0.80 (.60–1.08)
Control	12.71	6.08	Reference
Higher-transmission stratum			
MDA	51.85	14.20	0.85 (.63–1.15)
fMDA	44.02	28.32	0.97 (.73–1.29)
Control	79.93	54.78	Reference

Abbreviations: CI, confidence interval; fMDA, focal mass drug administration; HFCA, health facility catchment area; IRR, incidence rate ratio; MDA, mass drug administration.

^a^ Negative binomial model with random effect included at the health facility catchment area level, controlling for monthly total rainfall, enhanced vegetation index, and the previous month's case counts.

^b^ *P* < .05.

## DISCUSSION

We evaluated the short-term impact of 2 rounds of MDA or fMDA with DHAp against a control of no mass treatment in Southern Province, Zambia, in the context of strong prevention and surveillance efforts. To our knowledge this is the first CRCT assessing the impact of mass treatment interventions with DHAp in an African setting.

Large declines in study end points were observed in both intervention and control areas. Despite this, results during the first 5 months of the trial show 2 rounds of MDA with DHAp to have substantial impact on study end points in this setting. In areas of lower transmission, MDA was shown to reduce peak season child parasite infection prevalence from 8% before to <1% after MDA (87% decline). Individuals receiving MDA experienced a 3-fold decrease in cumulative infection incidence in lower-transmission areas (nonsignificant) and a significant 2-fold decrease in higher-transmission areas. These results are supported by monthly confirmed case incidence data derived from the health management information system.

Results from the trial were largely consistent across study end points. First, fMDA showed consistently less impact than MDA, with no results reaching statistical significance. Second, across all study end points, the effect size of 2 rounds of MDA was larger in lower-transmission than in higher-transmission areas.

It is likely that RDTs used during fMDA for treatment decisions still missed a substantial proportion of individuals with low-density parasite infections [9, 10]. Furthermore, by design, fMDA communities received only one-third of the total DHAp courses compared with MDA (Supplementary Table 1), leaving two-thirds of individuals in these areas without chemoprophylactic protection from piperaquine [13, 14] or from clearing subpatent infections. New HRP2 RDTs with much higher sensitivity to identify low-density infections are under development; these may render fMDA and mass testing and treatment strategies more effective in the future.

Although we identified many reports describing the short-term success of MDA campaigns under program settings [11, 12, 18–24], we identified only 2 randomized trials that assessed the impact of MDA in the African setting. Both studies evaluated MDA with artesunate plus sulphadoxin-pyrimethamine; neither showed a significant impact on malaria health outcomes in the short term [25, 26]. However, our results, especially in the lower-transmission setting, are consistent with mathematical modeling showing MDA-DHAp with the potential to significantly reduce malaria transmission to the point of interrupting it [27]. Our results show MDA-DHAp to have a much larger impact on malaria infection prevalence than found in previous studies of mass testing and treatment interventions with RDTs and AL [3–7], which is also consistent with simulation modeling from this area [27, 28].

We observed large decreases in malaria infection prevalence in children (by 73%) and confirmed malaria case incidence (by 43%) in control areas that coincided with mass treatment implemented in neighboring intervention areas. As a result, the expected detectable differences in these end points for comparing the mass treatment interventions to control was much lower than expected, resulting in lower statistical power than expected to detect significant differences [16]. It is unclear why such large declines in control areas occurred, but there are several potential explanations. First, mean monthly cumulative rainfall (117.5 mm) was lower during the rainy season just after the mass treatment rounds (January–April 2015) than during the previous rainy season in 2014 (137.3 mm), before the mass treatment rounds (Supplementary Figure 4). However, the rainfall in 2015 was very similar to that in 2012 (120.5 mm) before a parasite survey in the study area (conducted April–May) that showed malaria infection prevalence in children to be 35.6% [4], compared with an overall 7.9% in the parasite survey in 2015 (across lower- and higher-transmission areas). Although the decrease in rainfall probably played a role in the malaria decline in control areas between 2014 and 2015, it does not seem to fully explain it. We argue that, at least in part, the very high vector control coverage, strong surveillance, community diagnosis and case management, and reactive case detection across the study area may have played a role in the decline in malaria in control clusters.

Results of this trial should be interpreted in light of several limitations. First, the follow-up period of 5 months is insufficient to assess long-term trends in the malaria burden after mass treatment. However, the short follow-up period does allow measurement of short-term impact of mass treatment at driving the malaria burden down to preelimination levels. Two additional rounds of MDA and fMDA with DHAp were conducted in October 2015 and February 2016; we are continuing to collect data on primary end points and will report longer-term results once available. Second, because of the relatively small number of clusters (10 HFCAs in each treatment group in each transmission stratum), there was the potential for imbalance across treatment groups. However, all analyses of study end points also included models with potential confounders, stipulated a priori in the protocol [16]. It should be noted that effect estimates between crude and adjusted models were very similar, suggest that important confounding factors were balanced across treatment groups.

Third, we performed an intention-to-treat analysis; misclassification of the true exposure between MDA, fMDA, and control households owing to contamination across HFCA borders was possible. To mitigate misclassification and contamination, households within a 3-km buffer of HFCA boarders were excluded from the sampling frame for malaria parasite prevalence and cumulative infection incidence. Misclassification of HFCA into lower- and higher-transmission strata above and below 10% parasite prevalence was also possible. Fourth, the cumulative infection incidence analysis used RDTs. Although slides were collected and read for the cohort, positivity among slides was only a fraction of RDT positivity, probably a result of poor slide preparation among the 16 000 samples collected in the field. It is possible that RDTs were prone to yield more false-positive results than slides [29], especially in areas of low transmission. However, it is unlikely there were systematic differences in RDT false-positivity across treatment groups, so any bias would be limited. Finally, the follow-up survey was used to estimate household coverage of MDA and fMDA rounds based on respondent recall. It is hypothesized the much lower coverage of fMDA may have resulted from poorer recall, because treatment with DHAp in fMDA areas was only a fraction of that given during MDA. However, low coverage of the fMDA may have contributed to the observed lower impact compared with MDA.

Although there is no clear evidence linking MDA to antimalarial drug resistance [12], the potential exists for its development against component compounds of DHAp, especially when MDA is conducted at scale under routine program conditions. We used DHAp, the alternate first-line drug for uncomplicated malaria in Zambia, in part to mitigate resistance against the first line treatment AL [17]. We continue to monitor molecular markers of drug resistance and will report our findings when available.

In conclusion, 2 rounds of MDA had a substantial impact on malaria infection prevalence, cumulative infection incidence, and confirmed case incidence rates, especially in lower-transmission areas. It is important to highlight that the trial was conducted in an area of very high vector control, good surveillance, and improved access to case management, which we argue are prerequisite to implementing any MDA strategy in similar settings. In lower-transmission areas, infection prevalence among children during the peak transmission season went down to <1%, suggesting that transmission was reduced to a point where elimination may be possible. If these gains can be sustained by continued universal vector control coverage, strong surveillance, and case management, it may be possible to eliminate malaria from this area of Zambia.

## Supplementary Data

Supplementary materials are available at http://jid.oxfordjournals.org. Consisting of data provided by the author to benefit the reader, the posted materials are not copyedited and are the sole responsibility of the author, so questions or comments should be addressed to the author.

Supplementary Data
