# Supplementary material for: Short-term Impact of Mass Drug Administration With Dihydroartemisinin Plus Piperaquine on Malaria in Southern Province Zambia: A Cluster-Randomized Controlled Trial
Source: J Infect Dis. 2016 Dec 5;214(12):1831–9. doi: 10.1093/infdis/jiw416 (PMC5142084; doi:10.1093/infdis/jiw416)
Supplement: Supplementary Data [file supp_214_12_1831__index.html]

Supplementary Data 

# Short-term Impact of Mass Drug Administration With Dihydroartemisinin Plus Piperaquine on Malaria in Southern Province Zambia: A Cluster-Randomized Controlled Trial

## Supplementary Data

Supplementary Data

- Supplementary Data - Docx file
