# Supplementary material for: Short-term Impact of Mass Drug Administration With Dihydroartemisinin Plus Piperaquine on Malaria in Southern Province Zambia: A Cluster-Randomized Controlled Trial
Source: J Infect Dis. 2016 Dec 5;214(12):1831–9. doi: 10.1093/infdis/jiw416 (PMC5142084; doi:10.1093/infdis/jiw416)
Supplement: Supplementary Data [file supp_jiw416_jiw416supp.docx]

**Supporting information**

**Rainfall and EVI during the Trial**

Monthly total rainfall during the study period was similar between the treatment groups (SI Figure 4). Mean monthly cumulative rainfall during the 4-month rainy season (January – April) in 2015 following the mass treatments rounds was lower (117.5 mm) compared to this same period in 2014 (137.3 mm; t-value = -7.40, P<0.01) and 2013 (132.8 mm, t-value=-6.74, P<0.01), but was similar to 2012 (120.5 mm; t-value=-1.14, P=0.280, SI Figure 5). The decline in rainfall during the rainy season between 2014 and 2015 did not vary significantly by treatment group or transmission strata.

**SI Figure 1. Participant flow chart to determine antimalarial treatment regimen under MDA-DHAp. Women of reproductive age (WRA): those 15-49 years old; RDT: rapid diagnostic test; HF: health facility**

**SI Figure 2. Participant flow chart to determine antimalarial treatment regimen under fMDA-DHAp. Women of reproductive age (WRA): those 15-49 years old; RDT: rapid diagnostic test; HF: health facility**

**SI Figure 3. Flow chart for cohort enrollment and follow-up visit for both high and low transmission strata**

**SI Figure 4: Monthly cumulative rainfall in the study area August 2014 – July 2015, by treatment group**

**SI Figure 5. Mean monthly cumulative rainfall January – April 2012 – 2015 in the study area** The mean total rainfall for each 1 square kilometer pixel for each household in study area from January – April for the years 2012 – 2015 were totaled and presented in millimeters (mm). The plots show the minimum and maximum rainfall, median value, interquartile ranges and the frequency of the rainfall patterns (width of each plot).

**SI Figure 1: Participant flow chart to determine antimalarial treatment regimen under MDA-DHAp. Women of reproductive age (WRA): those 15-49 years old; RDT: rapid diagnostic test; HF: health facility**

**
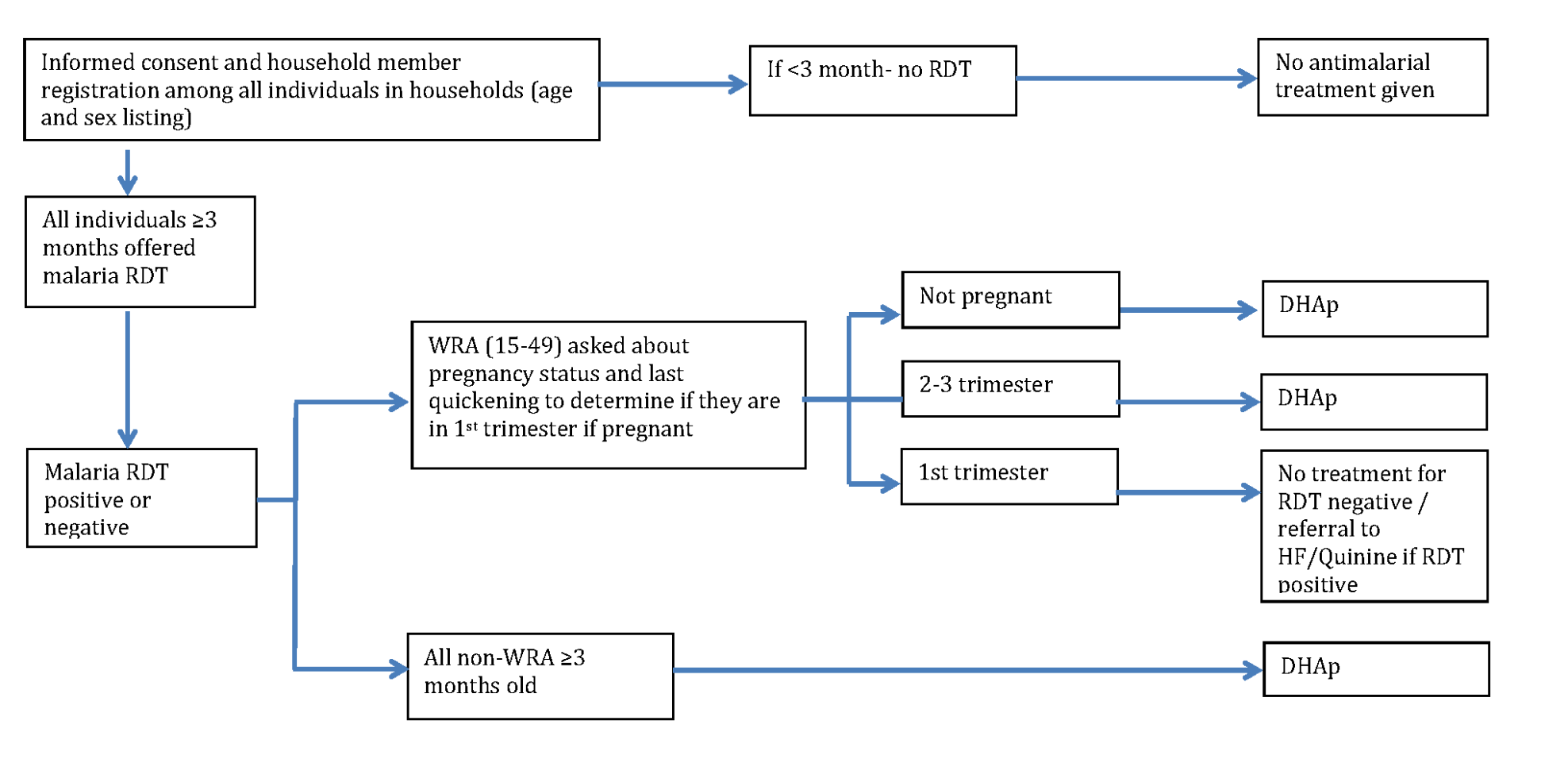
**

**SI Figure 2: Participant flow chart to determine antimalarial treatment regimen under fMDA-DHAp. Women of reproductive age (WRA): those 15-49 years old; RDT: rapid diagnostic test; HF: health facility**

**
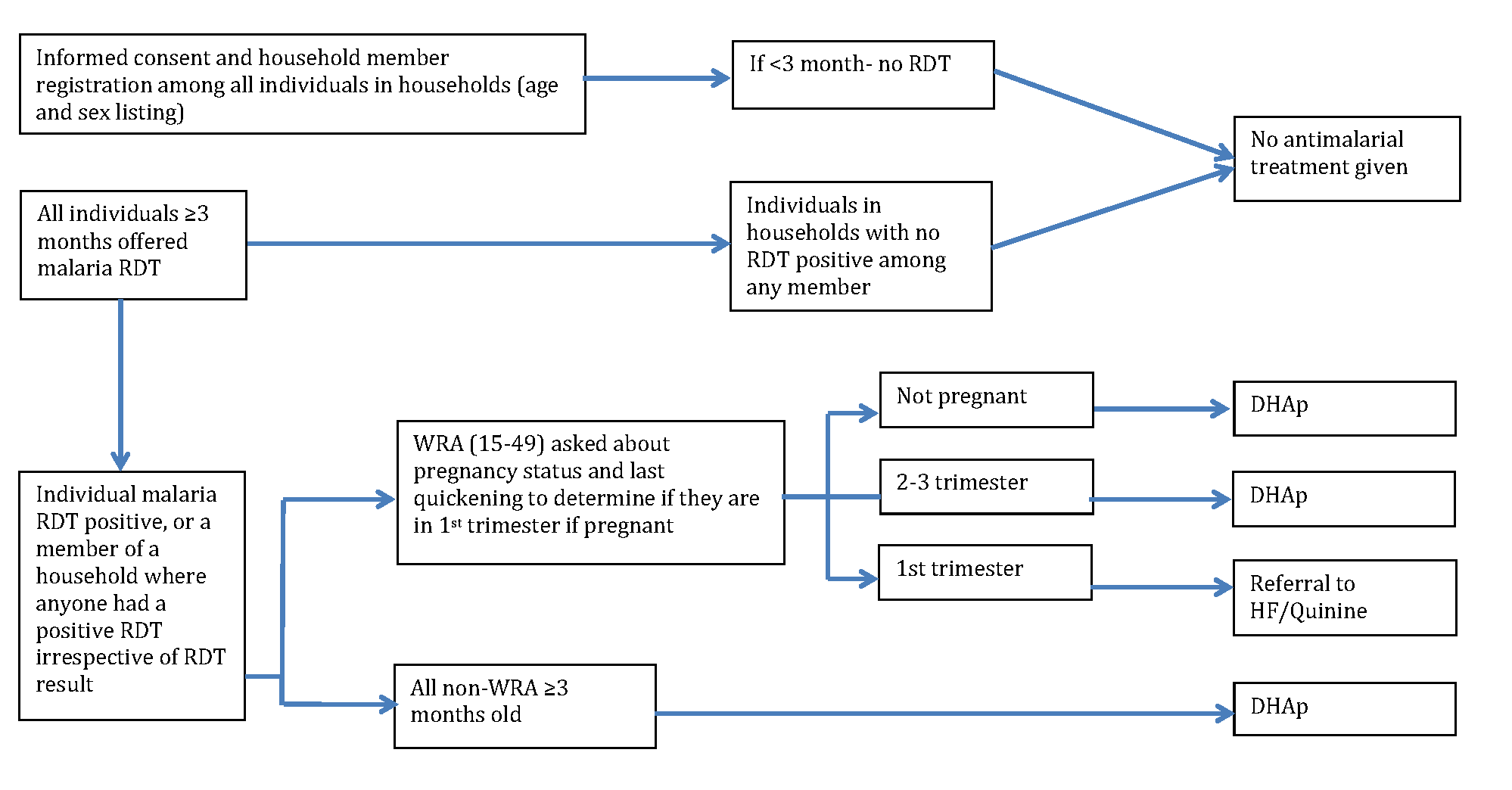
**

**SI Figure 3: Flow chart for cohort enrollment and follow-up visit for both high and low transmission strata**

Simple random sample of 2250 individuals (in 750 households) from sampling frame of 2014 baseline parasite survey

**Control group**

638 individuals (223 households) enrolled

***Enrollment***: During control RDT positivity data collection round 1 (December 2014): RDT positive receive AL

**Data collection**:

- RDT
- Microscopy
- DBS
- Questionnaire

***Follow-up***: Monthly through November 2015: RDT positive receive AL*

**Data collection**:

- RDT
- Blood blots
- Questionnaire
- 608 individuals completed at least 3 months of follow-up with a blood sample
- Loss-to-follow-up = 4.7%

731 individuals (248 households) enrolled

**fMDA group**

***Enrollment***: During fMDA round 1 (December 2014): members of household with RDT positive receive DHAp

**Data collection**:

- RDT
- Microscopy
- DBS
- Questionnaire

***Follow-up***: Monthly through November 2015: RDT positive receive AL*

**Data collection**:

- RDT
- Blood blots
- Questionnaire
- 627 individuals completed at least 3 months of follow-up with a blood sample
- Loss-to-follow-up = 14.2%

769 individuals (249 households) enrolled

***Enrollment***: During fMDA round 1 (December 2014): RDT positive and negative receive DHAp

**Data collection**:

- RDT
- Microscopy
- DBS
- Questionnaire

***Follow-up***: Monthly through November 2015: RDT positive receive AL*

**Data collection**:

- RDT
- Blood blots
- Questionnaire

**MDA group**

- 599 individuals completed at least 3 months of follow-up with a blood sample
- Loss-to-follow-up = 22.1%

*Pregnant women with a positive RDT in their first trimester will receive the standard of care treatment of uncomplicated malaria in Zambia consisting of oral quinine.

**SI Figure 4: Monthly cumulative rainfall in the study area August 2014 – July 2015, by treatment group**

**
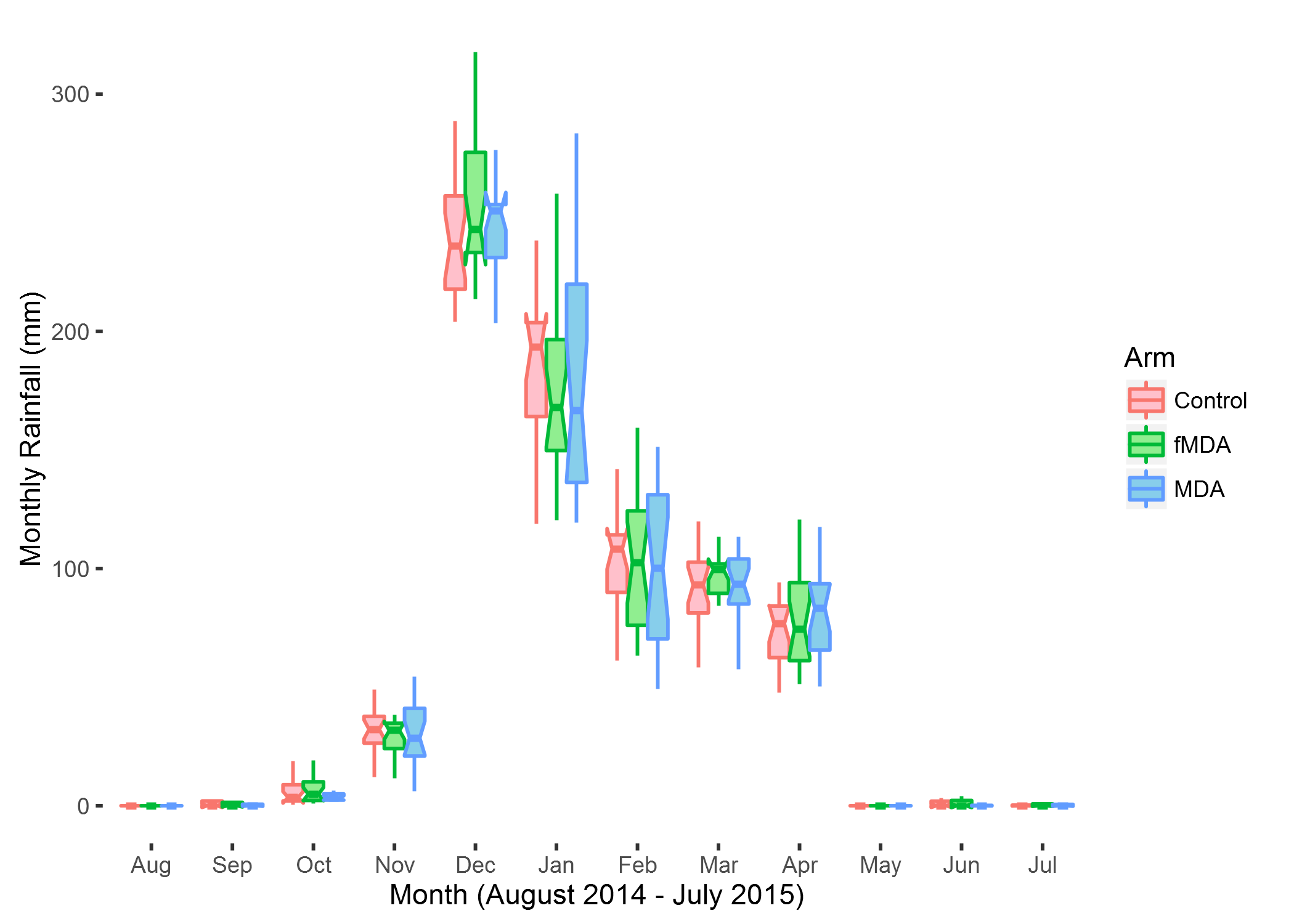
**

**SI Figure 5: Mean monthly cumulative rainfall January – April 2012 – 2015 in the study area**

**
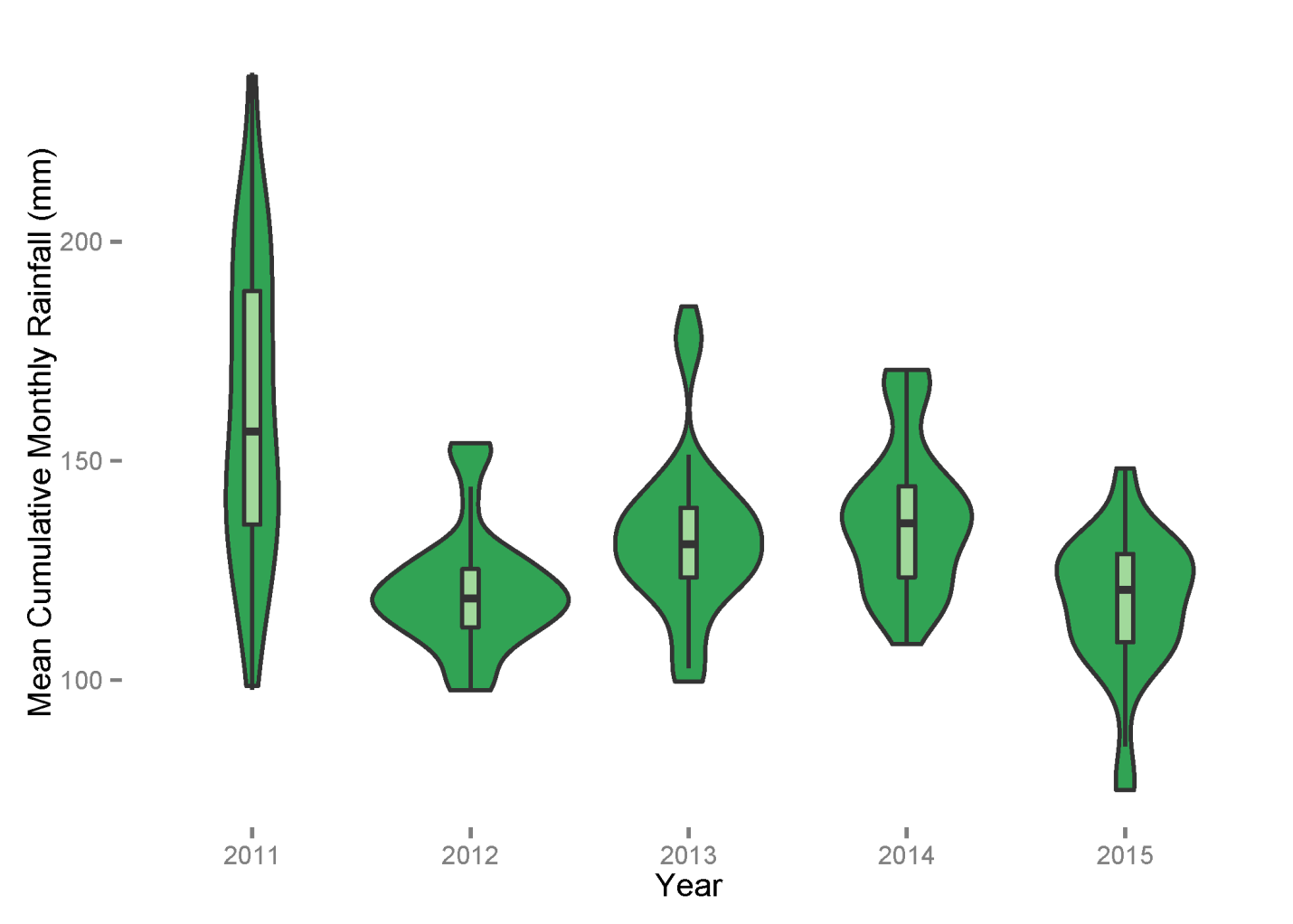
**

162.3 mm

120.5 mm

132.8 mm

137.3 mm

117.5 mm

**SI Table 1. Household coverage and DHAp adherence**

|  | **MDA** | | **fMDA** | |
| --- | --- | --- | --- | --- |
| **Indicator** | **Round 1** | **Round 2** | **Round 1** | **Round 2** |
| Total courses of DHAp administered | 78591 | 56620 | 25372 | 17092 |
| Total households visited | 18235 | 14584 | 17706 | 14610 |
| Household coverage (%)* | 88.1 | 72.0 | 62.5 | 54.0 |
| Individuals fully adherent to all doses of DHAp (%) | 84.6 | 80.6 | 91.5 | 80.6 |
| Individuals partially adherent to full course of DHAp (took ≥1 dose) (%) | 96.8 | 95.2 | 98.1 | 95.2 |
| Individuals who refused testing and/or treatment (%) | 0.18 | 0.20 | 0.73 | 0.37 |
| Individuals absent during testing and treatment (%) | 2.5 | 4.1 | 4.3 | 6.5 |

*Estimated for rounds 1 and 2 during the 2015 follow-up household survey

**SI Table 2. Baseline and follow-up malaria prevalence by microscopy among children <6 years old, as measured by the baseline and follow-up household surveys during the peak malaria transmission season April May 2014 and 2015**

|  | **Baseline (April-May 2014)** | | | | **Follow-up (April-May 2015)** | | | |
| --- | --- | --- | --- | --- | --- | --- | --- | --- |
| **Treatment group** | **n** | **Positive** | **%**  **(95 % CI)** | **Crude odds ratio**  **vs control**  **(95% CI)** | **n** | **Positive** | **%**  **(95 % CI)** | **Crude odds ratio**  **vs control**  **(95% CI) †** |
| **Lower transmission stata** |  |  |  |  |  |  |  |  |
| MDA | 543 | 19 | 3.50  (1.14 – 5.86) | 0.64  (0.27 – 1.55) | 373 | 0 | 0.00 | - |
| fMDA | 439 | 13 | 2.96  (0.33 – 5.59) | 0.53  (0.21 – 1.35) | 333 | 1 | 0.30  (0.00 – 1.02) | 0.18  (0.02 – 1.46) |
| Control | 436 | 24 | 5.50  (2.28 – 8.73) | Reference | 355 | 6 | 1.69  (0.13 – 3.25) | Reference |
| **Higher transmission strata** |  |  |  |  |  |  |  |  |
| MDA | 489 | 126 | 25.77  (14.11 – 37.42) | 0.75  (0.36 – 1.54) | 367 | 20 | 5.45  (0.48 – 10.42) | 0.76  (0.21 – 2.77) |
| fMDA | 519 | 159 | 30.64  (17.60 – 43.67) | 0.94  (0.46 – 1.92) | 305 | 14 | 4.59  (0.48 – 8.70) | 0.66  (0.18 – 2.47) |
| Control | 502 | 152 | 30.28  (19.12 – 41.44) | Reference | 332 | 22 | 6.63  (1.55 – 11.70) | Reference |

Standard errors for crude odds ratios are unadjusted to account for the CRCT study design using a random effect at the HFCA level.

*p < 0.05
